# Pembrolizumab-induced Thyroiditis, Hypophysitis and Adrenalitis: A Case of Triple Endocrine Dysfunction

**DOI:** 10.1210/jcemcr/luae200

**Published:** 2024-11-04

**Authors:** Silvia Rossi, Francesca Silvetti, Monia Bordoni, Alessandro Ciarloni, Gianmaria Salvio, Giancarlo Balercia

**Affiliations:** Endocrinology Clinic, Department of Clinical and Molecular Sciences, Polytechnic University of Marche, Ancona 60126, Italy; Endocrinology Clinic, Department of Clinical and Molecular Sciences, Polytechnic University of Marche, Ancona 60126, Italy; Endocrinology Clinic, Department of Clinical and Molecular Sciences, Polytechnic University of Marche, Ancona 60126, Italy; Endocrinology Clinic, Department of Clinical and Molecular Sciences, Polytechnic University of Marche, Ancona 60126, Italy; Endocrinology Clinic, Department of Clinical and Molecular Sciences, Polytechnic University of Marche, Ancona 60126, Italy; Endocrinology Clinic, Department of Clinical and Molecular Sciences, Polytechnic University of Marche, Ancona 60126, Italy

**Keywords:** thyroiditis, hypophysitis, adrenalitis, pembrolizumab, melanoma, PD1, checkpoint inhibitors

## Abstract

Immune checkpoint inhibitor drugs can trigger autoimmune endocrine reactions as a known side effect. Several cases of immunotherapy-induced autoimmune endocrinopathies have been described, but multiple sequential endocrine toxicities are a rare occurrence. A 39-year-old patient with metastatic melanoma started adjuvant therapy with pembrolizumab. One month later he presented with asymptomatic thyrotoxicosis and, within several weeks, overt hypothyroidism, for which he started levothyroxine therapy. Subsequently the patient developed central adrenal insufficiency due to probable hypophysitis, and steroid replacement therapy was started. Pembrolizumab therapy was then discontinued. After a few months, a full recovery of pituitary function was observed, but primary adrenal insufficiency occurred, requiring additional fludrocortisone therapy. The described clinical case is a very uncommon case of triple endocrinological toxicity from immunotherapy. The clinical and biochemical manifestations of immunotherapy-induced endocrinopathies can be variable and atypical; therefore, it is necessary to pay special attention to any clue of hormonal dysfunction.

## Introduction

Immune checkpoint inhibitor (ICI) drugs can trigger autoimmune endocrine reactions, and several cases of immunotherapy-induced autoimmune endocrinopathies have been described in the literature [1]. However, multiple endocrine toxicities are rare: the case we are going to describe shows a triple endocrinological toxicity from pembrolizumab and the possibility that toxicities are reversible.

## Case Presentation

On August 2021, a 39-year-old male underwent surgical removal of a skin melanoma on the right arm, which was subsequently classified as pT1b pN1a (< 1 mm) M0 (stage IIIA). After being informed about risks and benefits of adjuvant immunotherapy in relation to the disease stage, the patient decided in favor of this type of treatment.

Thus, he underwent adjuvant therapy with pembrolizumab 100 mg IV administration once every 21 days, with the first administration in January 2022.

Routine blood tests were performed before the second administration and revealed lowered levels of TSH, increased levels of free T3 and free T4 (Table 1), without hallmark signs and symptoms of hyperthyroidism, and therapy with methimazole was initiated. The second dose of pembrolizumab was then administered in February 2022. In the meantime, further investigations showed a well-preserved anterior pituitary function. The neck ultrasound showed an irregular and hypervascularized thyroid gland with absence of evident nodularities.

**Table 1.** Blood parameters in 2022

Blood parameters	Normal value	2/15	3/8	3/23	5/24	5/27	8/5
ACTH	8-60 pg/mL (1.76-13.2 pmol/L)					7.88 pg/mL (1.7 pmol/L)	
Cortisol	5-23 µg/dL (137.9-634.4 nmol/L)			6.92 µg/dL (190.8 nmol/L)	0.69 µg/dL (19 nmol/L)	0.54 µg/dL (14.8 nmol/L)	0.8 µg/dL (22 nmol/L)
TSH	0.350-3.5 µIU/mL (0.350-3.5 IU/L)	<0.0015 µIU/mL (<0.0015 IU/L)	19.616 µIU/mL (19.616 IU/L)	89.082 µIU/mL (89.082 IU/L)	60.394 µIU/mL (60.394 IU/L)	11.727 µIU/mL (11.727 IU/L)	35.891 µIU/mL (35.891 IU/L)
FT3	2,3-4 pg/mL (3.5-6.1 pmol/L)	9.1 pg/mL (13.9 pmol/L)	3.2 pg/mL (4.9 pmol/L)	3.08 pg/mL (4.7 pmol/L)	3.8 pg/mL (5.8 pmol/L)	2.6 pg/mL (3.9 pmol/L)	2.48 pg/mL (3.8 pmol/L)
FT4	0,54-1,24 pg/mL (0.69-1.59 pmol/L)	0.94 pg/mL (1.21 pmol/L)	<0.25 pg/mL (<0.32 pmol/L)	0.28 pg/mL (0.36 pmol/L)	0.91 pg/mL (1.17 pmol/L)	0.85 pg/mL (1.09 pmol/L)	0.9 pg/mL (1.15 pmol/L)
FSH	1.4-16 mIU/mL (1.4-16 IU/L)				1.59 mIU/mL (1.59 IU/L)	4.55 mIU/mL (4.55 IU/L)	
LH	1.3-9 mIU/mL (1.3-9 IU/L)				8.39 mIU/mL (8.39 IU/L)	6.01 mIU/mL (6.01 IU/L)	
Total testosterone	2.7-11 ng/mL (9.3-38.1 nmol/L)				1.11 ng/mL (3.8 nmol/L)	1.98 ng/mL (6.8 nmol/L)	
PRL	2-17 ng/mL (2-17 ng/mL)				20.56 ng/mL (20.56 ng/mL)	6.9 ng/mL (6.9 ng/mL)	
GH	<3 ng/mL (<3 µg/L)					0.11 ng/mL (0.11 µg/L)	0.185 ng/mL (0.185 µg/L)
IGF-1	80-250 ng/mL (10.4-326 nmol/L)						121.3 ng/mL (15.8 nmol/L)
IGFBP3	3.2-6.6 µg/mL (32000-66000 ng/mL)					5.55 µg/mL (555 000 ng/mL)	
DHEAS	106-464 μg/dL (2.88-12.60 μmol/L)					23 μg/dL (0.62 μmol/L)	

Abbreviations: DHEAS, dehydroepiandrosterone; FT3, free T3; FT4, free T4; IGFBP3, IGF-binding protein 3; PRL, prolactin.

After 14 days, the patient repeated the blood tests, which showed overt hypothyroidism that was confirmed after 3 weeks of discontinuation of methimazole, so levothyroxine therapy was started. Notably, at that time cortisol levels at the lower limits of normal range were detected. In the following months, 3 doses of pembrolizumab were administered.

In May 2022, the patient was brought to the emergency department of the Augusto Murri Hospital of Fermo (Marche, Italy) with fever, constipation, dizziness, asthenia, headache, and nausea; blood tests revealed secondary adrenal insufficiency, hypogonadotropic hypogonadism, hyponatremia, slightly increased prolactin levels, and elevated TSH, with normal free fractions of thyroid hormones and positivity of thyroid peroxidase antibodies and antithyroglobulin antibodies (Table 2).

**Table 2.** Autoimmune panel

Blood parameters	Normal values	5/24/2022	5/17/2023
Ab anti-TPO	<9 IU/mL (< 9 IU/mL)	26.6 IU/mL (26.6 IU/mL)	
Ab anti-Tg	< 4 IU/mL (< 4 IU/mL)	118 IU/mL (118 IU/mL)	
Ab anti-21 hydroxylase	< 0.4 IU/mL (<0.4 IU/mL)		< 0.23 IU/mL (<0.23 IU/mL)

Abbreviations: Ab anti-21 hydroxylase, anti-21-hydroxilase antibodies; Ab anti-Tg, antithyroglobulin antibodies; Ab anti-TPO, thyroid peroxidase antibodies.

The patient was thus admitted to the internal medicine unit, where IV saline hydration and glucocorticoid replacement therapy were administered. Furthermore, a normalization of prolactin levels was observed.

During the hospitalization, magnetic resonance imaging (MRI) without contrast of the pituitary gland showed no morphological abnormalities; in addition, a total body computed tomography scan with contrast did not show any adrenal abnormalities.

The patient was discharged in June 2022 with a diagnosis of adrenal insufficiency due to probable iatrogenic hypophysitis and autoimmune thyroiditis, both related to pembrolizumab therapy, which was finally discontinued.

## Diagnostic Assessment

In February 2023, the patient underwent andrological evaluation at our outpatient clinics (endocrinology clinic, AOU delle Marche, Italy) for secondary infertility resulting from erectile dysfunction and decreased libido. Moreover, he complained of profound asthenia, blepharospasm, and fasciculation of the lower extremities despite high doses of cortisone acetate (62.5 mg/day). The patient presented in excellent general clinical condition, with normal vital parameters and physical examination, including skin color. The only detectable feature was the presence of fasciculations in the lower limbs.

Semen analysis revealed sperm concentration at the lower limit of the normal range and reduced sperm motility; sperm culture and detection of *Chlamydia trachomatis* and *Mycoplasma* in the urine were negative. Scrotal ultrasonography revealed testicular volume at the lower limit of the normal range and slightly inhomogeneous testicular echo structure, with scattered microcalcifications.

To further investigate the case, the patient was admitted to our inpatient clinic: blood tests revealed persistence of elevated TSH levels, increased ACTH levels, low levels of adrenal androgens, high renin levels with low aldosterone levels, and hypogonadotropic hypogonadism (Table 3).

**Table 3.** Blood parameters in 2023

Blood parameters	Normal value	2/1	5/17	12/9
ACTH	8-60 pg/mL (1.76-13.2 pmol/L)	320.9 pg/mL (70.6 pmol/L)	1268 pg/mL (279.2 pmol/L)	1090 pg/mL (240 pmol/L)
Cortisol	5-23 µg/dL (137.9-634.4 nmol/L)	0.21 µg/dL (5.7 nmol/L)	0 µg/dL (0 nmol/L)	10.3 µg/dL (284.1 nmol/L)
TSH	0.350-3.5 µIU/mL (0.350-3.5 IU/L)	46.659 µIU/mL (46.659 IU/L)	0.777 µIU/mL (0.777 IU/L)	0.228 µIU/mL (0.228 IU/L)
FT3	2.3-4 pg/mL (3.5-6.1 pmol/L)	3.8 pg/mL (5.8 pmol/L)	4 pg/mL (6.1 pmol/L)	
FT4	0.54-1.24 pg/mL (0.69-1.59 pmol/L)	0.6 pg/mL (0.77 pmol/L)	1.14 pg/mL (1.46 pmol/L)	0.75 pg/mL (0.96 pmol/L)
FSH	1.4-16 mIU/mL (1.4-16 IU/L)	5.87 mIU/mL (5.87 IU/L)	3.2 mIU/mL (3.2 IU/L)	2.69 mIU/mL (2.69 IU/L)
LH	1.3-9 mIU/mL (1.3-9 IU/L)	3.92 mUI/mL (3.92 UI/L)	7.1 mUI/mL (7.1 UI/L)	3.54 mUI/mL (3.54 UI/L)
Total testosterone	2.7-11 ng/mL (9.3-38.1 nmol/L)	2.08 ng/mL (7.2 nmol/L)	4.76 ng/mL (16.5 nmol/L)	3.11 ng/mL (10.7 nmol/L)
SHBG	14.5-48.4 nmol/L (14.5-48.4 nmol/L)		24.2 nmol/L (24.2 nmol/L)	22 nmol/L (22 nmol/L)
PRL	2-17 ng/mL (2-17 ng/mL)	12.36 ng/mL (12.36 ng/mL)	20.1 ng/mL (20.1 ng/mL)	
GH	<3 ng/mL (<3 µg/L)	0.32 ng/mL (0.32 µg/L)	0.07 ng/mL (0.07 µg/L)	
IGF1	80-250 ng/mL (10.4-326 nmol/L)	197 ng/mL (25.7 nmol/L)	116 ng/mL (15.1 nmol/L)	
IGFBP3	3.2-6.6 µg/mL (32000-66000 ng/mL)		7.36 µg/mL (736 000 ng/mL)	
DHEAS	106-464 μg/dL (2.88-12.60 μmol/L)		0.26 µg/mL (0.7 mcmol/L)	25 μg/dL (0.68 μmol/L)
17OHP	0.5-2.1 ng/mL (1.5-6.3 nmol/L)		0.63 ng/mL (1.9 nmol/L)	
Androstenedione	0.4-2.6 ng/mL (1.3-9 nmol/L)		<0.3 ng/mL (<1 nmol/L)	0.3 ng/mL (1 nmol/L)
Renin	4.4-46.10 mcUI/mL (4.4-46.10 mUI/mL)	245.7 mcUI/mL (245.7 mUI/mL)	127.30 mcUI/mL (127.3 mUI/mL)	28.86 mcUI/mL (28.86 mUI/mL)
Aldosterone	35-300 pg/mL (0.09-0.8 nmol/L)	18 pg/mL (0.04 nmol/L)	27 pg/mL (0.07 nmol/L)	
Sodium	135-145 mEq/L (135-145 mEq/L)	139 mEq/L (139 mEq/L)		
Potassium	3.5-5 mEq/L (3.5-5 mEq/L)	4 mEq/L (4 mEq/L)		

Abbreviations: 17OHP, 17α-hydroxyprogesterone; DHEAS, dehydroepiandrosterone; FT3, free T3; FT4, free T4; IGFBP3, IGF-binding protein 3; PRL, prolactin.

Given the symptoms and blood test suggestive for primary adrenal insufficiency, despite the negativity of anti-21 hydroxylase antibodies (Table 2), mineralocorticoid replacement therapy was started and glucocorticoid replacement therapy continued.

Brain MRI with contrast medium showed no abnormalities of the pituitary gland, and thyroid ultrasound showed a slightly inhomogeneous gland, with no nodules in context.

The patient was discharged in May 2023 with a diagnosis of pembrolizumab-induced primary adrenal insufficiency, primary hypothyroidism, and previous partial hypopituitarism.

## Treatment

The patient was initially treated with methimazole (at a dose of 20 mg/day). Once overt hypothyroidism was established, replacement therapy was started with levothyroxine, first in tablet formulation at a dose of 75 mcg alternating with 100 mcg/day. In the following months, the patient continued pembrolizumab therapy with 3 administrations, and the dose of levothyroxine was optimized (100 mcg/day).

At the emergency department of the Augusto Murri Hospital of Fermo, the patient was treated with IV hydrocortisone (50 mg × 3/day for 2 days), then switched to oral cortisone acetate (starting with 50 mg upon awakening and 12.5 mg at lunchtime).

Considering the serious adverse events, and the early stage of melanoma, therapy with pembrolizumab was discontinued (last administration was in April 2022).

At the endocrinological follow-up evaluation, due to the persistence of elevated TSH values, the dose of levothyroxine was gradually increased up to 175 mcg/day, switching to a liquid formulation. Glucocorticoid replacement therapy was also modified, switching from cortisone acetate to modified-release hydrocortisone 20 mg upon awakening. Due to the clinical worsening characterized by asthenia and fasciculations, the glucocorticoid replacement therapy was again modified by resuming cortisone acetate therapy (50 mg upon awakening and 12.5 mg at lunchtime).

Following the visit to our center (endocrinology clinic, AOU delle Marche, Italy), fludrocortisone therapy was introduced and the dosage of cortisone acetate was reduced (25 mg upon awakening and 12.5 mg in the early afternoon), then switched to modified-release hydrocortisone (25 mg upon awakening).

## Outcome and Follow-up

After the introduction of fludrocortisone therapy and adjustments of glucocorticoid substitution therapy, the patient experienced clinical improvement as well as a normalization of plasma renin levels.

Semen analysis was repeated after 3 months and revealed an improvement in seminal parameters.

Six months later, blood tests revealed low TSH levels with normal free T3 and free T4 so levothyroxine therapy was titrated (175 mcg/day alternating with 150 mcg/day, liquid formulation).

## Discussion

**Figure 1.**
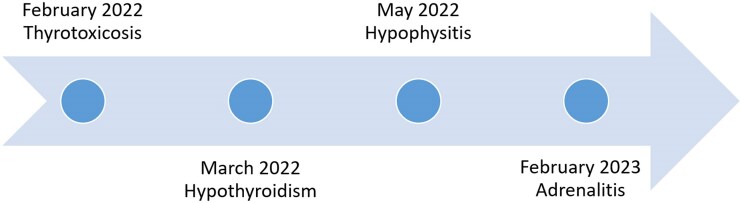
Timeline of endocrinological toxicities.

This case report is remarkable as there are no cases of triple endocrine dysfunction following adjuvant treatment with pembrolizumab (see Fig. 1), with the 3 conditions arising in succession, in the literature.

The patient first developed thyroid toxicity; indeed, chronic lymphocytic thyroiditis is a common adverse effect of pembrolizumab, and it can manifest as hypothyroidism, thyrotoxicosis, or a combination of both. It more commonly develops in cases showing previous positivity of antithyroid antibodies and a family history of autoimmune thyroid disease [2] and in patients treated with programmed death 1 (PD1) inhibitors than cytotoxic T-lymphocyte-associated protein 4 inhibitors [3]. Whether our patient had thyroiditis prior to the initiation of pembrolizumab therapy is unknown; nevertheless, this became evident as thyrotoxicosis first and as hypothyroidism after methimazole therapy, respectively about 5 and 8 weeks after the start of ICI therapy, with the timing consistent with that reported in the literature [3].

Despite the unavailability of TSH receptor antibody levels at the first evaluation, the peculiar succession of events is suggestive of an autoimmune lymphocytic thyroiditis characterized by thyrotoxicosis at its onset and a later phase of hypothyroidism. The first condition was mistaken for Basedow-Graves disease; only rare cases of Basedow-Graves disease have been related to immunotherapy in the literature [4].

Subsequently, the patient developed central glucocorticoid axis deficiency. Pituitary dysfunction is a well-known adverse event in patients treated with ICIs [5], and pembrolizumab-related hypophysitis has been described in depth in the literature [6]. Albeit rare, its occurrence is more often associated with cytotoxic T-lymphocyte-associated protein 4 inhibitors, rather than PD1 inhibitors [1].

The diagnosis of pituitary deficit was supported by suggestive clinical and biochemical evidence, as well as by the presence of a possible iatrogenic cause, with timing consistent with that described in the literature, that is, approximately 4 months from the beginning of ICI therapy [1, 3]. Other etiologies were excluded through a detailed evaluation of the patient's medical history; indeed, he had no history of pituitary surgery or pituitary macroadenomas.

The diagnosis of hypophysitis is the most plausible despite the negative signs at MRI, including the absence of a thickened pituitary stalk. It is important to highlight that a negative pituitary MRI does not exclude the diagnosis of immunotherapy-related hypophysitis [7]. This is especially true in PD1-induced hypophysitis cases [8]. In the absence of a histopathological confirmation, a definite diagnosis is impossible to achieve. However, the typical pattern of autoimmune hypophysitis including isolated glucocorticoid deficits without other axis deficits [6] supported the hypothesis of pembrolizumab-related hypophysitis.

The gonadal deficit is most likely explained by functional hypogonadism due to the patient's general conditions. Indeed, the seminal alterations were minimal and were not confirmed at a second analysis, as a result of the recovery of the gonadal axis.

The patient then developed primary adrenal insufficiency with mineralocorticoid deficiency but negative 21 hydroxylase antibodies. This condition is well explained by the persistence of asthenia despite high-dose steroid replacement therapy.

The third condition that the patient developed was insidious to recognize and diagnose, since primary adrenal insufficiency is remarkably rare in patients on ICI monotherapy [1]. For this reason, there are no unambiguous data regarding the timing of the onset of the condition in relation to the initiation of ICI [3]. Moreover, negativity for 21 hydroxylase antibodies is a confounding factor. It is key to mention that rare cases of autoimmune adrenalitis are antibody-negative [9, 10], and antibody assays should be repeated as clinical conditions change in patients treated with ICIs.

The reversibility of pituitary dysfunction—evidenced in our case by increased ACTH levels and mineralocorticoid deficiency—highlighted 1 of the possible patterns of progression of this condition, which has never been described before. Indeed, the onset of adrenalitis allowed for a reassessment of pituitary hormone tests, which showed a resolution of the pituitary deficit.

## Learning Points

In our opinion, this clinical case shows unique aspects never previously described that conclude in the following guidelines:

- Place clinical suspicion of multiple endocrinologic toxicities in patients undergoing immunotherapy if suggestive symptomatology is present, including onset some time after the last administration and in case of single immunotherapy.
- In questionable cases, pay close attention to any clue of hormonal dysfunction and retest the patient if there are significant clinical changes that are not explained by the previously diagnosed conditions.
- Wait for spontaneous recovery of the gonadotropic axis if there is a chance that the damage is transient and attributable to the patient's general health status.

## Data Availability

Data sharing is not applicable to this article as no datasets were generated or analyzed during the current study.
