# Correction to: “Pembrolizumab-induced Thyroiditis, Hypophysitis and Adrenalitis: A Case of Triple Endocrine Dysfunction”

**DOI:** 10.1210/jcemcr/luaf254

**Published:** 2025-10-08

In the above-named article by Rossi S, Silvetti F, Bordoni M, Ciarloni A, Salvio G, and Balercia G (*JCEM Case Reports*. 2024, 2(11); doi: 10.1210/jcemcr/luae200), there was an error in Table 1 and Table 3.

In the originally published article, in Table 1, under the column “Normal value,” in the row “DHEAS,” “1-4.5 µg/mL (2.7-12.2 mcmol/L)” has been corrected to read, 106.0-464.0 μg/dL (2.88-12.60 μmol/L).” In Table 1, under the column “5/27,” in the row “DHEAS,” the value “23 µg/mL (62.4 mcmol/L)” has been corrected to read, “23.0 µg/dL (0.62 µmol/L).”

In the originally published article, in Table 3, under the column “Normal value,” in the row “DHEAS,” “1-4.5 µg/mL (2.7-12.2 mcmol/L)” has been corrected to read, “106.0-464.0 μg/dL (2.88-12.60 μmol/L).” In Table 3, under the column “12/9,” in the row “DHEAS,” the value “25 µg/mL (67.8 mcmol/L)” has been corrected to read, “25.0 µg/dL (0.68 µmol/L).”

The article has been corrected online.

doi: 10.1210/jcemcr/luae200

